# Chronic Ethanol Exposure during Adolescence Increases Voluntary Ethanol Consumption in Adulthood in Female Sprague Dawley Rats

**DOI:** 10.3390/brainsci10120900

**Published:** 2020-11-24

**Authors:** Antoniette M. Maldonado-Devincci, Cheryl L. Kirstein

**Affiliations:** 1 Department of Psychology, College of Health and Human Sciences, North Carolina Agricultural and Technical State University, Greensboro, NC 27411, USA; 2 Department of Psychology, Cognitive and Neurosciences, University of South Florida, Tampa, FL 33620, USA; kirstein@usf.edu; 3 Molecular Pharmacology & Physiology, University of South Florida College of Medicine, Tampa, FL 33612, USA

**Keywords:** alcohol, adolescence, sex differences, female, rat, consumption, blood ethanol concentrations, LORR

## Abstract

Early alcohol use is a major concern due to the dramatic rise in alcohol use during adolescence. In humans, adolescent males and females consume alcohol at equivalent rates; however, in adulthood males are more likely to consume harmful levels of alcohol. In animal models, the long-term dose-dependent and sex-dependent effects of alcohol exposure during adolescence have not been readily assessed relative to exposure that is initiated in adulthood. The purpose of the present set of experiments was to determine if adolescent exposure to chronic ethanol would predispose male and female rats to greater ethanol intake in adulthood when compared to animals that were not exposed to chronic ethanol exposure until early adulthood. Male and female rats were chronically administered 0.75 g/kg or 1.5 g/kg ethanol or saline for 21 days during adolescence (postnatal day (PND) 30–50) or adulthood (PND 60–80). All rats subsequently underwent 14-days of abstinence (PND 51–64 or PND 81–94, respectively). Finally, all rats were given 30-min daily access to saccharin-sweetened ethanol or saccharin alone from PND 65–80 for adolescent-exposed rats and PND 95–110 for adult-exposed rats. Exposure to 0.75 g/kg ethanol did not alter ethanol or saccharin intake in adolescent-exposed or adult-exposed rats, regardless of sex. In contrast, chronic exposure to the higher 1.5 g/kg dose during adolescence increased ethanol intake in adulthood in female rats. However, there was no change in saccharin intake in animals exposed to 1.5 g/kg ethanol during adolescence or adulthood, regardless of sex. Additionally, there were no clear age- and ethanol-dependent changes in duration of loss of righting reflex and blood ethanol concentrations to a challenge administration of a higher dose of ethanol. The results of the present set of experiments indicate chronic exposure to a high dose of ethanol during adolescence in female rats did indeed predispose rats to consume more ethanol in adulthood. Given that these effects were only observed in adolescent-exposed female rats, these results support a unique vulnerability to the long-term consequences of adolescent ethanol exposure in female rats, an effect that is not merely mediated by the sweetener used in the ethanol solution.

## 1. Introduction

Adolescence is the time period in which most alcohol experimentation and the development of addiction begins [1]. It has been estimated that up to 50% of adolescents report current use of alcohol by the time they enter the 12th grade [2]. Escalated rates of alcohol drinking during adolescence can be attributed to increases in risk-taking, novelty-seeking, and social behavioral motivations [3,4]. On average, adults drink more often than adolescents, but adolescents tend to drink more per occasion [5]. Elevated intake of alcohol later in life in both humans and rodents has been reported following early and repeated heavy alcohol consumption during adolescence (for a review see [4]). Drinking before age 15 in humans is associated with a several-fold higher drinking likelihood in adulthood compared to those that waited longer to begin drinking alcohol [6]. Consequently, the impact of adolescent episodic heavy or binge drinking is important to understand its effects on prolonged alcohol drinking behaviors [2].

Recent work in humans shows that male and female adolescents report overall similar levels of alcohol use, but females who begin drinking at an early age drink more than other relevant age groups [7,8]. In rodent work, sex differences in voluntary alcohol drinking have been observed, beginning in adolescence [9]. These data indicate the age of initiation of alcohol use can be a significant predictor of higher alcohol and other substance abuse later in life [7,10,11] and highlight important sex differences based on adolescent alcohol use that may influence alcohol use disorders later in life.

Alcohol has biphasic effects on behavior. Adult rats show sedation, motor incoordination, and aversion to high doses of ethanol [12,13,14,15]. In contrast, low ethanol doses produce stimulatory effects of locomotor activity [16] in adult rats. Adolescent rodents are less sensitive to the sedative and hypnotic effects of ethanol and more sensitive to the rewarding properties of ethanol compared to adult rats [12,13,14,17]. We have previously shown that binge ethanol exposure during adolescence results in (1) increased ethanol consumption in adulthood in both male and female rats and that (2) adolescent male rats consume more ethanol than adult male rats [18,19,20]. In contrast, other work has shown that rats given voluntary access to unsweetened ethanol beginning in adolescence and extending into adulthood (PND 28–90) showed no differences from rats that began voluntarily consuming ethanol beginning in adulthood (PND 71–90; [21]). Forced periadolescent (PND 30–40) exposure to ethanol vapor for 12 h a day also did not enhance sucrose sweetened ethanol drinking in adulthood (>PND 92; [22]). Together, these data indicate that the type and pattern of ethanol exposure can induce differences in later voluntary ethanol consumption following exposure during adolescence.

Loss of righting reflex (LORR) has long been used as an index in sensitivity to acute intoxication to a high dose of ethanol [12,13,14,23,24,25]. In studies conducted in mice, no sex differences in duration of LORR were observed [26,27]. Adolescent ethanol-exposed mice showed shorter duration for LORR when collapsed across sex [27] or no difference in duration of LORR in female mice [26] when compared to adult ethanol-exposed mice. However, when blood ethanol concentrations (BECs) were assessed at the recovery of LORR, adolescent C57BL/6J mice showed no age or sex differences and adolescent DBA/2J mice showed higher BECs at recovery compared to adult DBA/2J mice [27]. Work conducted in rats showed that adolescent rats are less sensitive to the sedative and hypnotic effects of ethanol and show faster ethanol metabolism compared to adult rats [12,13,14,24,25]. Together, these data indicate that sex-dependent long-term changes in the assessment of LORR are needed.

Early exposure to ethanol during adolescence may predispose those individuals to greater ethanol intake in adulthood. Therefore, the present set of experiments aimed to assess the dose-dependent long-term effects of chronic ethanol exposure on voluntary ethanol intake in adolescent-exposed vs. adult-exposed male and female rats. Specifically, we aimed to assess (1) the age-related differences in subsequent voluntary ethanol intake during adolescence or adulthood and (2) changes in responsivity to a sedating dose of ethanol following chronic exposure to 0.75 or 1.5 g/kg ethanol during adolescence or adulthood in male and female rats. We expected exposure to both doses of ethanol to increase later ethanol consumption in adolescent-exposed compared to adult-exposed male and female rats. 

## 2. Materials and Methods

### 2.1. Subjects

Male and female Sprague–Dawley rats, offspring of established breeding pairs, (Harlan Laboratories, Indianapolis, IN, USA) at the University of South Florida, Tampa were used in the present studies. A total of 340 rats (male *n* = 153 and female *n* = 187) were used in the consumption and LORR studies and 192 (male *n* = 95 and female *n* = 97) rats were used for the BECs time course analysis experiment. Litters were sexed and culled to 10 pups per litter on postnatal day (PND) 1, with the day of birth designated as PND 0. Each litter was culled to six males and four females whenever possible. Extra pups were used in other ongoing experiments. Pups remained with their respective dams until PND 21, when pups were weaned and pair-housed with same-sex littermates. Animals were maintained on a 12:12 h light: dark cycle, in a temperature and humidity-controlled vivarium. Animals were allowed free access to food and water in their home cage. Rats were randomly assigned to conditions of 0.75 or 1.5 g/kg ethanol or an isovolumetric administration of saline. No more than one pup per litter was placed in any given condition. In all respects, maintenance and treatment of the animals was within the guidelines for animal care by the National Institutes of Health under University of South Florida Institutional Animal Care and Use Committee (IACUC) (M3592) approved protocols. 

### 2.2. Procedure

**Figure 1:**
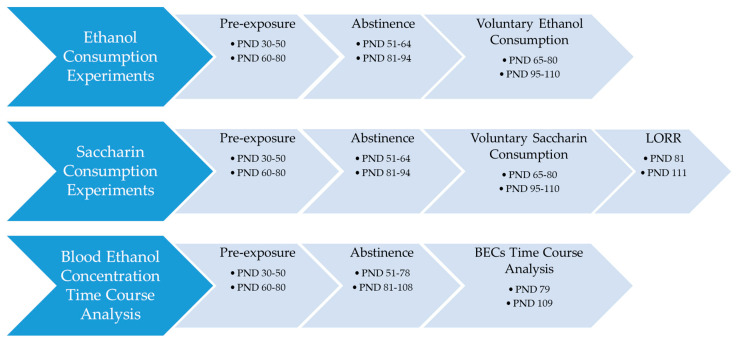
Timeline for each set of experiments.

A timeline for each set of experiments is shown in Figure 1.

Handling: Adolescent animals were gently handled from PND 28–29 and adult animals from PND 58–59 for five min each.

Pre-exposure: For 21 consecutive days, adolescent (PND 30 to 50) and adult (PND 60 to 80) rats received daily intraperitoneal (i.p.) injections of 0.75 or 1.5 g/kg ethanol (17% volume/volume (*v*/*v*) in saline; Pharmco Aaper, Shelbyville, KY, USA) or saline (0.9% NaCl; Fisher Scientific, Hampton, NH, USA). Saline was administered isovolumetric to the ethanol dose, so there was a control group for each ethanol dose. 

Abstinence: All rats remained ethanol abstinent from PND 51–64 for adolescent-exposed and PND 81–94 for adult-exposed rats. During this time, all rats were left undisturbed in the colony, except for regular cage maintenance, which occurred twice per week.

Voluntary Intake of Saccharin Sweetened Ethanol or Saccharin Alone: Ethanol consumption and saccharin consumption were conducted in separate sets of experiments. Adolescent-exposed (PND 65–80) and adult-exposed (PND 95–110), rats were assessed daily for voluntary consumption of a sweetened ethanol solution (0.5% saccharin (weight/volume (*w*/*v*))/10% ethanol (*v*/*v*) or saccharin alone (0.5% *w*/*v* saccharin; Fisher Scientific, Hampton, NH, USA) using a limited access two-bottle choice paradigm. The choice was always between sweetened ethanol and water or between saccharin and water. For the ethanol consumption experiments, fresh bottles were presented to all rats daily with one bottle containing a saccharin/ethanol solution and the other bottle containing tap water. For the saccharin consumption experiments, fresh bottles were presented daily with the choice between one bottle containing the saccharin only solution and the other both containing tap water. To avoid the development of a side preference, the placement of the saccharin/ethanol or saccharin only and water bottles were alternated daily. Bottles were weighed to the nearest 0.1 g before and after the 30 min access period and the difference indicated ethanol consumed as grams of ethanol per kilogram of body weight (g/kg). Spillage was accounted for by placing the bottles in an unoccupied holding cage and the difference calculated for spillage was subtracted from the daily difference calculated for each rat.

Each day of the two-bottle choice testing period, rats were transported to the laboratory, weighed, and habituated for 30 min. Rats were then given 30 min access to two bottles with one containing the sweetened ethanol or saccharin (described above) and the other containing water. After the 30 min access period, both bottles were removed and weighed again, and all rats were presented with the original water bottle. Rats were then left in the experimental room for an additional 60 min, after which they were returned to the colony. This procedure was repeated each day between 0900–1200 h during the light cycle.

Loss of Righting Reflex and Blood Ethanol Concentrations: On PND 81 (adolescent-exposed) or PND 111 (adult-exposed), all rats from the saccharin consumption experiments were challenged with 3.0 g/kg ethanol and assessed for LORR according to previously published methods [28]. Briefly, rats were administered an i.p. injection of ethanol and placed in a supine position. The duration of time it took for the rat to right itself on all four paws was recorded. Any rat that did not lose its righting reflex was not included in the analysis. Upon recovery of LORR, blood was collected from the saphenous vein and used to measure blood ethanol concentrations. All blood samples were centrifuged at 5000 g and serum collected to analyze blood ethanol concentrations using the AM1 Analox Alcohol Analyzer (Analox Instruments, Lunenburg, MA, USA). During processing some of the samples were lost due to technical difficulties. 

Blood Ethanol Concentration Time Course Analysis: In a separate experiment, rats were exposed to saline or 0.75 g/kg from PND 30–50 (adolescent-exposed) or from PND 60–80 (adult-exposed). All rats underwent abstinence for 28 days from PND 51–78 for adolescent-exposed rats and PND 81–108 for adult-exposed rats. Rats were challenged with 1.75 g/kg ethanol (i.p.) on PND 79 for adolescent-exposed rats and PND 109 for adult-exposed rats. Trunk blood samples were collected after 5, 15, 30, and 60 min post-injection in different rats. All blood samples were processed as outlined above.

### 2.3. Design and Analyses

Ethanol consumption/preference, and saccharin consumption data were analyzed separately for each dose and sex with a three factor mixed-model design ANOVA with Age (2; Adolescent, Adult) and Exposure (2; Saline, Ethanol) as between-subjects factors and Day (collapsed across two-day intervals) as a within-subjects factor. LORR and BEC data were analyzed separately for each sex using a three factor between subjects ANOVA with Age (2; Adolescent, Adult), Exposure (2; Saline, Ethanol), and Dose (2, 0.75 or 1.0 g/kg). For the blood ethanol concentration time course analysis data were analyzed separately for each sex. Data were matched for time and analyzed using a three-factor mixed model design ANOVA for Age (2; Adolescent, Adult), Exposure (2; Saline, Ethanol), and Time (5, 15, 30, 60 min). For any analyses that showed a Geisser–Greenhouse epsilon value of 0.75 or lower, the df was adjusted and reported in the F tables below. In the presence of significant interactions, Tukey’s and Sidak’s multiple comparisons post hoc tests were used where appropriate. 

## 3. Results

### 3.1. Ethanol Consumption and Preference

#### 3.1.1. Ethanol Consumption

**Figure 2:**
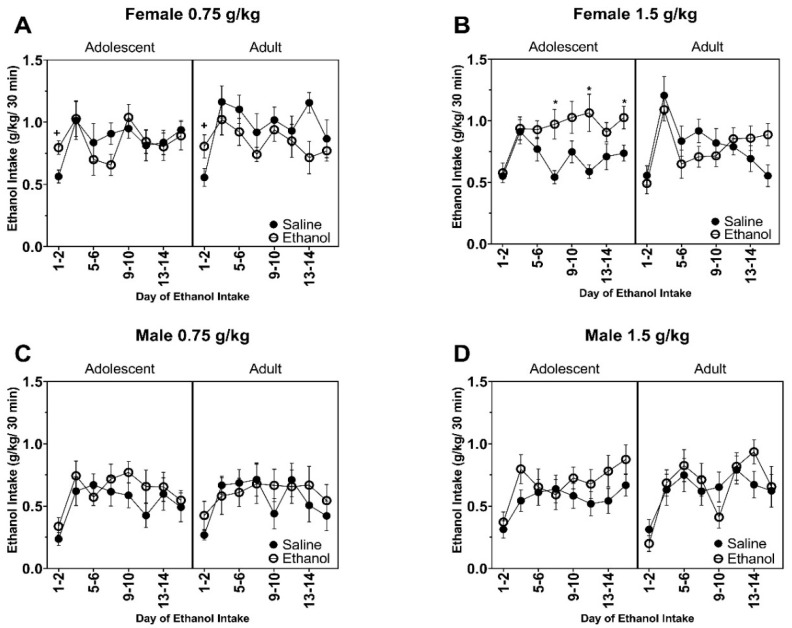
Data are presented as ethanol consumed (g/kg) for the 30 min session. Female rats chronically exposed to (**A**) 0.75 g/kg and (**B**) 1.5 g/kg. Male rats chronically exposed to (**C**) 0.75 g/kg and (**D**) 1.5 g/kg. + indicates ethanol is significantly greater than saline when collapsed across age. * indicates ethanol-exposed significantly greater than ethanol-exposed.

All F values for ethanol consumption data are reported in Table 1 and Table 2. Overall, female rats that were chronically exposed to 0.75 g/kg ethanol showed increased voluntary ethanol consumption at the first time point in both adolescent-exposed or adult-exposed rats (Figure 2A) as supported by a Day by Exposure interaction (F (7, 301) = 2.19, *p* < 0.05) and a main effect of Day (F (7, 301) = 4.81, *p* < 0.0005). Chronic exposure to 1.5 g/kg ethanol in female rats increased voluntary ethanol consumption in adulthood in adolescent-exposed, but not adult-exposed rats (Figure 2B) as supported by an Age by Exposure (F (1, 42) = 8.06, *p* < 0.01) and Day by Exposure (F (7, 293) = 2.11, *p* < 0.05) interactions and main effects of Exposure (F (1, 42) = 6.30, *p* < 0.02) and Day (F (7, 293) = 8.16, *p* < 0.0001). The Age by Exposure by Day interaction failed to reach statistical significance. Data for the female rats were separated by age for post hoc analyses. Adolescent ethanol-exposed female rats showed increased ethanol consumption at days 7–8, 11–12, and 15–16 of the two-bottle choice paradigm. There were no changes in voluntary ethanol intake in the male rats following pre-exposure to 0.75 g/kg (Figure 2C) or 1.5 g/kg (Figure 2D) ethanol during adolescence or adulthood. However, ethanol consumption did change across days in male rats pre-exposed to 0.75 g/kg ethanol as supported by a main effect of Day (F (7, 245) = 4.87, *p* < 0.0005) and to 1.5 g/kg ethanol as supported by a main effect of Day (F (7, 252) = 7.137, *p* < 0.0001). All other main effects and interactions failed to reach statistical significance in the male rats. 

#### 3.1.2. Ethanol Preference

**Figure 3:**
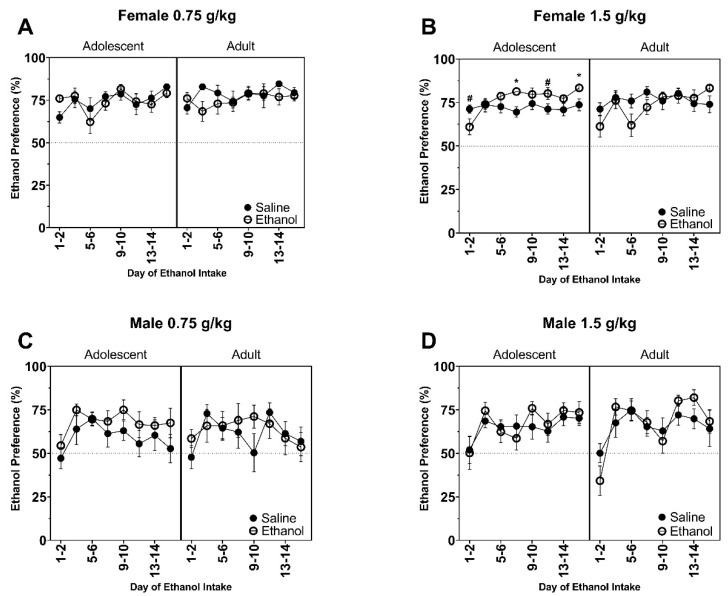
Data are presented as preference for ethanol ((ethanol (mL)/ ethanol + water (mL)) × 100) for the 30 min session. Female rats chronically exposed to (**A**) 0.75 g/kg and (**B**) 1.5 g/kg. Male rats chronically exposed to (**C**) 0.75 g/kg and (**D**) 1.5 g/kg. * indicates ethanol-exposed significantly greater than ethanol-exposed. # indicates *p* = 0.06 for ethanol-exposed rats to have increased ethanol preference compared to saline-exposed rats.

All F values for ethanol preference data are reported in Table 3 and Table 4. Chronic pre-exposure to 0.75 g/kg ethanol did not alter ethanol preference later in adulthood in adolescent-exposed or adult-exposed female rats (Figure 3A), but ethanol preference did change across days as supported by a main effect of Day (F (7, 301) = 2.50, *p* < 0.05). Similar to ethanol consumption, preference for ethanol was increased in female rats that were pre-exposed to 1.5 g/kg during adolescence on Day 7–8 and 15–16 (Figure 3B), as supported by an Age by Exposure (F (1, 42) = 6.34, *p* < 0.02) and Day by Exposure (F (7, 294) = 2.56, *p* < 0.02) interactions and main effect of Day (F (7, 294) = 4.42, *p* < 0.001). A trend for increased preference in adolescent ethanol-exposed female rats showed higher preference on Day 1–2 and 11–12 (*p* = 0.06). Preference for ethanol varied across days in male rats pre-exposed to 0.75 g/kg (Figure 3C) as supported by a main effect of Day (F (7, 249) = 2.882, *p* < 0.05) and 1.5 g/kg (Figure 3D) as supported by a main effect of Day (F (7, 252) = 8.27, *p* < 0.0001). 

#### 3.1.3. Saccharin Consumption

**Figure 4:**
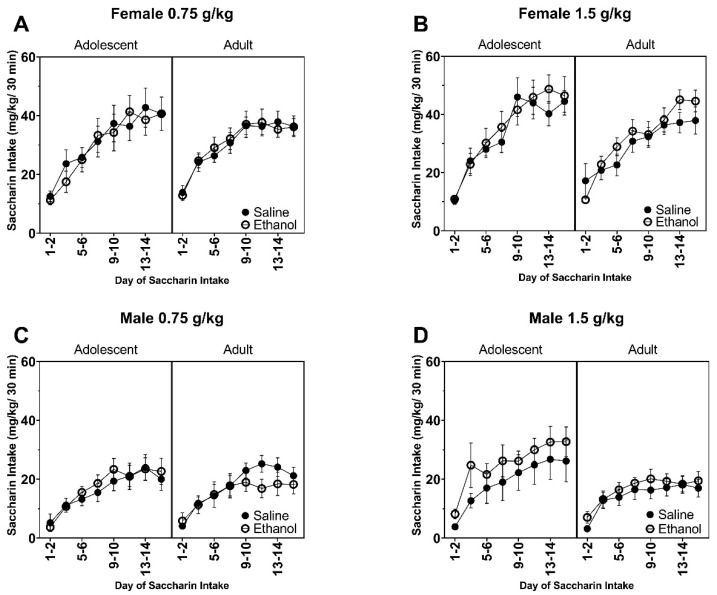
Data are presented as saccharin consumed (mL/kg) for the 30 m in session. Female rats chronically exposed to (**A**) 0.75 g/kg and (**B**) 1.5 g/kg. Male rats chronically exposed to (**C**) 0.75 g/kg and (**D**) 1.5 g/kg.

All F values for saccharin consumption data are reported in Table 5 and Table 6. In female rats pre-exposed to 0.75 g/kg ethanol, saccharin consumption in adulthood increased across the 16 days of the two-bottle choice paradigm (Figure 4A) as supported by a main effect of Day (F (4.3, 190.8) = 48.85, *p* < 0.0001). For female rats that were pre-exposed to ethanol, there was a change in saccharin consumption across days (Figure 4B) as supported by a Day by Age interaction (F (7, 301) = 2.25, *p* < 0.05) and main effect of Day (F (4.7, 202.3) = 58.28, *p* < 0.0001). However, post hoc analyses between ages failed to reveal any significant differences at any one time point. For male rats that were pre-exposed to 0.75 g/kg ethanol (Figure 4C), there was an increase in saccharin consumption across days as supported by a main effect of Day (F (4.4, 149.5) = 31.93, *p* < 0.0001). There were no other significant differences in saccharin consumption in the male rats exposed to the 0.75 g/kg dose. For male rats that were pre-exposed to the 1.5 g/kg dose of ethanol (Figure 4D), there was an overall higher preference for saccharin in the adolescent-exposed male rats compared to the adult-exposed male rats as supported by a Day by Age interaction (F (7, 245) = 2.06, *p* < 0.05) and main effects of Age (F (1, 35) = 4.17, *p* < 0.05) and Day (F (3.5, 121.9) = 23.19, *p* < 0.0001).

#### 3.1.4. Loss of Righting Reflex and Blood Ethanol Concentrations

**Figure 5:**
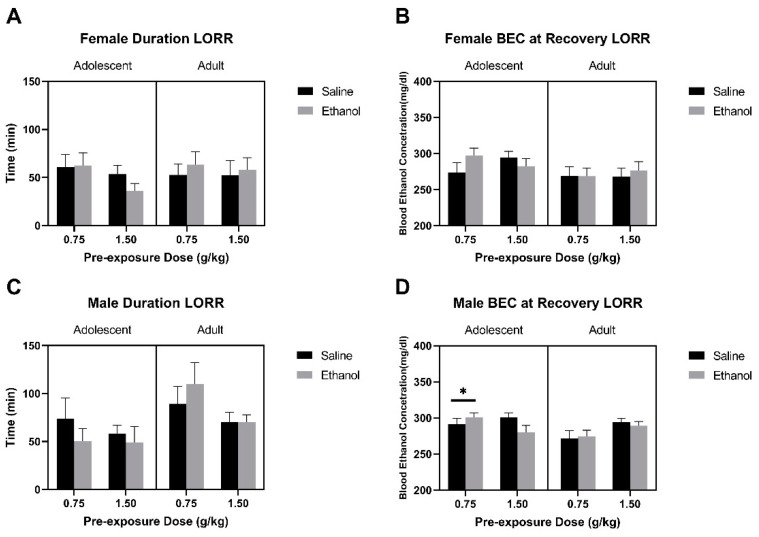
Data are presented as duration (min) of loss of righting reflex (Panels **A**,**C**) and blood ethanol concentration (mg/dL) at recovery of LORR (Panels **B**,**D**) following administration of 3.0 g/kg i.p. for male and female rats exposed to 0.75 or 1.5 g/kg ethanol or saline during adolescence or adulthood. * indicates adolescent is significantly higher than adult.

All F values for LORR and BECs at recovery of LORR data are reported in Table 7 and Table 8. Female rats that were pre-exposed to either dose of ethanol and consumed saccharin in adulthood did not show any differences in the duration for the loss of righting reflex (Figure 5A). However, there was a trend for higher BECs at the recovery of righting reflex in the adolescent-exposed female rats compared to the adult-exposed female rats (Figure 5B) as supported by trend for Age (F (1, 77) = 3.81, *p* = 0.055). Regardless of pre-exposure dose, male adolescent-exposed rats showed overall shorter duration for LORR compared to adult-exposed male rats (Figure 5C) as supported by a main effect of Age (F (1, 64) = 6.19, *p* < 0.02). There was a trend for male rats exposed to 1.5 g/kg ethanol to have a lower duration for LORR compared to those exposed to 0.75 g/kg (Dose: F (1, 64) = 3.02, *p* = 0.08). Regardless of pre-exposure to saline or ethanol, male rats that were exposed to 0.75 g/kg during adolescence had higher blood ethanol concentrations compared to male rats that were exposed during adulthood (Figure 5D) as supported by a Dose by Age interaction (F (1, 54) = 4.74, *p* < 0.05) and a main effect of Age (F (1, 54) = 3.99, *p* = 0.05). Additionally, there was a trend for a Dose by Exposure interaction (F (1, 54) = 2.91, *p* = 0.09). 

#### 3.1.5. Blood Ethanol Concentration Time Course Analysis

**Figure 6:**
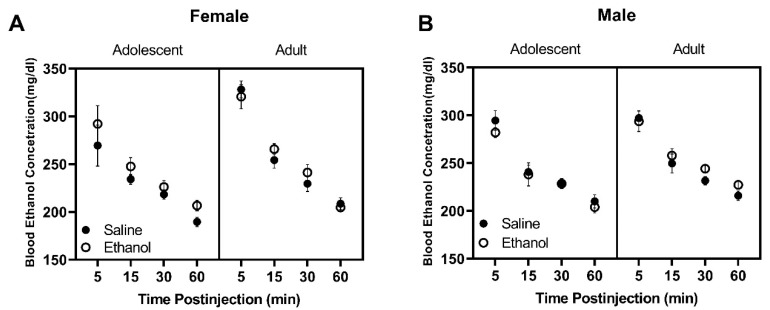
Data are presented as blood ethanol concentration (mg/dL) in response to 1.75 g/kg i.p. ethanol across time in female (Panel **A**) and male (Panel **B**) rats pre-exposed to saline or 0.75 g/kg ethanol during adolescence or adulthood.

All F values for the BECs time course analysis data are reported in Table 9. Regardless of pre-exposure to saline or ethanol, female rats showed age-dependent changes in BECs across time in response to a challenge injection of 1.75 g/kg ethanol (Figure 6A) as supported by a Time by Age interaction (F (3, 60) = 3.27, *p* < 0.05) and main effects of Age (F (1, 21) = 9.54, *p* < 0.01) and Time (F (1.6, 31.1) = 93.46, *p* < 0.0001). However, post hoc analyses failed to reveal any significant differences at any given time point. Male rats showed differences in BECs between age and across time (Figure 6B) as supported by a main effect of Time (F (2.2, 43) = 104.90, *p* < 0.0001) and Age (F (1, 21) = 5.65, *p* < 0.05). 

## 4. Discussion

Data from the present set of experiments suggest that exposure to the higher dose of ethanol during adolescence led female rats to greater consumption of sweetened ethanol in adulthood. Because this effect was only observed in the adolescent ethanol-exposed female rats, these results support a unique susceptibility to the long-term consequences of adolescent ethanol exposure in female rats. We did not observe any robust changes in blood ethanol concentrations in response to a challenge injection of ethanol. 

Low dose ethanol exposure did not readily alter chronic ethanol consumption in adulthood or responsiveness to a sedating dose of ethanol in later adulthood. However, we previously showed that chronic exposure to the low dose of ethanol during adolescence altered basal dopamine levels in adulthood in male rats [29]. Low doses of ethanol impaired the memory of an appetitively-motivated odor discrimination in adolescents [23]. Therefore, it is quite possible that the low dose ethanol exposure has effects that are independent of changes in ethanol consumption and ethanol sensitivity, such as memory and cognition. Therefore, future work should use other behavioral measures to assess changes in responsiveness to chronic exposure to a low dose of ethanol.

Previous research suggests that higher ethanol doses have a greater impact compared to lower doses in both age groups [14]. In the present work chronic exposure to the 1.5 g/kg dose of ethanol to female rats during adolescence increased voluntary ethanol consumption in adulthood compared to the saline adolescent-exposed controls. When comparing adolescent ethanol-exposed rats to adult ethanol-exposed rats, there were no differences in ethanol consumption at any given day, but in general adolescent ethanol-exposed rats showed greater ethanol consumption compared to adult ethanol-exposed rats when data were averaged across the entire drinking period (data not shown). When compared to adult rats, adolescent rats have shown higher ethanol intake over a 2 h session [30]. However, the adolescent rats consumed the majority of ethanol during the first 30 min, whereas the adult rats show a more evenly spread pattern of ethanol consumption over the 2 h duration [30]. We observed similar findings in response to chronic exposure to the high dose of ethanol in the adolescent ethanol-exposed female rats following 30 min access to ethanol using a two-bottle choice drinking paradigm. In future work, we should determine ethanol consumption for longer than 30 min.

Much work has been conducted using different models of intermittent ethanol exposure during adolescence. Many different models of ethanol exposure, including gavage, intraperitoneal injections, forced consumption, drinking in the dark, and others, have shown alterations in drinking, anxiety, and behavioral flexibility in different strains of rats [19,20,31,32,33,34]. Involuntary ethanol exposure during adolescence to 2.0 g/kg via intraperitoneal injections decreased ethanol consumption in adulthood in male Wistar rats [32]. We previously published work showing that chronic intermittent ethanol exposure via gavage increased sweetened ethanol intake in adulthood [20]. We have also recently shown that systemically administered ethanol induces greater deficits in behavioral flexibility in rats compared to intragastric gavage [35]. Therefore, the present findings extend our previous work in that we measured the effects of chronic ethanol exposure via intraperitoneal injections to determine long-term changes in ethanol consumption and ethanol pharmacokinetics in adolescent and adult male and female rats.

One of the strengths of the present work is that we demonstrate the effects of adolescent compared to adult ethanol exposure on voluntary saccharin consumption for each sex and both doses. This allowed us to determine the specificity in the change in ethanol consumption in adulthood in the female rats exposed to the high dose of ethanol during adolescence. In the saccharin consumption experiments, we did not observe any age-related or dose-related changes in limited access voluntary saccharin consumption. Therefore, we demonstrated that the sweetener used in the ethanol solution was not the driving factor for changes in ethanol consumption. 

There are clear developmental differences in the effects of ethanol. Adolescent rats are less sensitive to the sedative effects of ethanol compared to adult rats [13] and show tolerance to the hypnotic effects of ethanol [12,24]. Adolescent rats also show evidence of faster ethanol metabolism compared to adult rats [14,25]. All of this may predict long-term responsiveness to subsequent ethanol exposure after a washout period. We did not observe differences in BECs in the adolescent-exposed compared to adult-exposed rats when assessing changes in LORR following ethanol pre-exposure and saccharin consumption. Future work should assess the impact of the development of tolerance to chronic ethanol immediately following pre-exposure in adolescence and later in adulthood, especially in female rats. 

Our work is similar to previous work conducted in C57BL/6J mice [27], where there were no ethanol-specific differences in BECs following LORR. However, the current work assessed duration of LORR in adult rats following ethanol exposure during adolescence or adulthood and subsequent saccharin drinking. It is possible that the prolonged abstinence period following ethanol pre-exposure impacted any differences that might have been observed immediately following pre-exposure. Indeed, recent work showed that adult male and female mice that were exposed to chronic ethanol vapor inhalation showed shorter duration of LORR compared to air-exposed mice [26]. Another strength of the present work was the detailed 60 min time course analysis for BECs following administration of the challenge dose of ethanol. Future work should assess the (1) duration of LORR and BECs at recovery from LORR immediately following pre-exposure to saline and ethanol and (2) longer time points for the BECs time course analysis to determine age- and sex-dependent effects of chronic ethanol pre-exposure on these measures. 

## 5. Conclusions

The data from the present work may be indicative of a threshold for the dose of chronic ethanol early during adolescence, which could alter subsequent ethanol drinking later in life in female rats. The higher ethanol dose produced changes in both absolute ethanol intake (g/kg) and preference in female rats, but not male rats. For female rats, there were no age differences in the hypnotic response to the sedative dose of ethanol following ethanol pre-exposure. However, adolescent-exposed male rats tended to show a shorter duration of LORR compared to adult-exposed rats. In addition, regardless of pre-exposure to saline or ethanol, male rats exposed to the low dose of ethanol had higher average BECs for adolescent-exposed compared to adult-exposed rats. These data suggest that changes in chronic exposure to a high dose of ethanol during adolescence induces changes in ethanol consumption in female rats. For male rats, there is evidence that pre-exposure to ethanol may induce changes in the sedative response to an ethanol challenge in later adulthood. Overall, the sweetener used in the present set of experiments was not solely responsible for the change in ethanol consumption in the female rats. These data highlight the unique vulnerability in female rats to the lasting impact of ethanol exposure during adolescence, inducing greater ethanol intake in adulthood. 

## Figures and Tables

**Table 1:** Table for ANOVA results for female ethanol consumption data.

Female Ethanol Consumption	0.75 g/kg Exposure		1.5 g/kg Exposure	
	F Value	*p* Value	F Value	*p* Value
Day	(7, 301) = 4.81	0.0002	(7, 294) = 8.15	<0.0001
Age	(1, 43) = 1.02	0.32	(1, 42) = 0.21	0.65
Exposure	(1, 43) = 1.51	0.23	(1, 42) = 5.9	0.02
Age by Exposure	(1, 43) = 0.99	0.33	(1, 42) = 7.61	0.009
Age by Day	(7, 301) = 0.96	0.46	(7, 294) = 1.70	0.11
Exposure by Day	(7, 301) = 2.19	0.03	(7, 294) = 2.06	0.04
Age by Exposure by Day	(7, 301) = 0.51	0.82	(7, 294) = 1.64	0.12

**Table 2:** Table for ANOVA results for male ethanol consumption data.

Male Ethanol Consumption	0.75 g/kg Exposure		1.5 g/kg Exposure	
	F Value	*p* Value	F Value	*p* Value
Day	(7, 245) = 4.87	0.0002	(7, 252) = 7.14	<0.0001
Age	(1, 35) = 0.00	0.99	(1, 36) = 0.26	0.6165
Exposure	(1, 35) = 1.93	0.17	(1, 36) = 2.43	0.1281
Age by Exposure	(1, 35) = 0.16	0.69	(1, 36) = 1.16	0.2886
Age by Day	(7, 245) = 0.59	0.76	(7, 252) = 1.60	0.1371
Exposure by Day	(7, 245) = 0.66	0.70	(7, 252) = 0.88	0.5242
Age by Exposure by Day	(7, 245) = 0.47	0.85	(7, 252) = 0.62	0.7421

**Table 3:** Table for ANOVA results for female ethanol preference data.

Female Ethanol Preference	0.75 g/kg Exposure		1.5 g/kg Exposure	
	F Value	*p* Value	F Value	*p* Value
Day	(7, 301) = 2.50	0.03	(7, 294) = 4.4	0.0005
Age	(1, 43) = 1.30	0.26	(1, 42) = 0.11	0.74
Exposure	(1, 43) = 0.62	0.44	(1, 42) = 0.57	0.45
Age by Exposure	(1, 43) = 0.50	0.48	(1, 42) = 6.34	0.016
Age by Day	(7, 301) = 1.18	0.31	(7, 294) = 0.75	0.63
Exposure by Day	(7, 301) = 1.61	0.13	(7, 294) = 2.56	0.01
Age by Exposure by Day	(7, 301) = 0.60	0.76	(7, 294) = 1.30	0.25

**Table 4:** Table for ANOVA results for male ethanol preference data.

Male Ethanol Preference	0.75 g/kg Exposure		1.5 g/kg Exposure	
	F Value	*p* Value	F Value	*p* Value
Day	(7, 245) = 2.89	0.01	(7, 252) = 8.27	<0.0001
Age	(1, 35) = 0.085	0.77	(1, 36) = 0.06	0.81
Exposure	(1, 35) = 2.23	0.14	(1, 36) = 0.44	0.51
Age by Exposure	(1, 35) = 0.65	0.42	(1, 36) = 0.001	0.97
Age by Day	(7, 245) = 0.64	0.72	(7, 252) = 1.98	0.06
Exposure by Day	(7, 245) = 0.62	0.74	(7, 252) = 0.93	0.48
Age by Exposure by Day	(7, 245) = 0.66	0.71	(7, 252) = 0.66	0.70

**Table 5:** Table for ANOVA results for female saccharin consumption data.

Female Saccharin Consumption	0.75 g/kg Exposure		1.5 g/kg Exposure	
	F Value	*p* Value	F Value	*p* Value
Day	(4.3, 190.8) = 48.85	<0.0001	(4.7, 202.3) = 58.28	0.0001
Age	(1, 44) = 0.008	0.93	(1, 43) = 1.22	0.27
Exposure	(1, 44) = 0.01	0.91	(1, 43) = 0.52	0.48
Age by Exposure	(1, 44) = 0.043	0.84	(1, 43) = 0.02	0.88
Age by Day	(7, 308) = 1.22	0.29	(7, 301) = 2.25	0.03
Exposure by Day	(7, 308) = 0.69	0.68	(7, 301) = 1.60	0.13
Age by Exposure by Day	(7, 308) = 0.35	0.93	(7, 301) = 0.52	0.82

**Table 6:** Table for ANOVA results for male saccharin consumption data.

Male Saccharin Consumption	0.75 g/kg Exposure		1.5 g/kg Exposure	
	F Value	*p* Value	F Value	*p* Value
Day	(4.4, 149.5) = 31.93	<0.0001	(3.5, 121.9) = 23.19	<0.0001
Age	(1, 34) = 0.014	0.9061	(1, 35) = 4.17	0.048
Exposure	(1, 34) = 0.06	0.8067	(1, 35) = 1.63	0.21
Age by Exposure	(1, 34) = 0.53	0.4733	(1, 35) = 0.38	0.54
Age by Day	(7, 238) = 0.28	0.9613	(7, 245) = 2.06	0.048
Exposure by Day	(7, 238) = 0.87	0.5273	(7, 245) = 0.15″	0.99
Age by Exposure by Day	(7, 238) = 0.83	0.5602	(7, 245) = 0.54	0.81

**Table 7:** Table for ANOVA results for loss of righting reflex data.

Duration Loss of Righting Reflex	Female		Male	
	F Value	*p* Value	F Value	*p* Value
Dose	(1, 78) = 1.2	0.27	(1, 64) = 3.02	0.09
Age	(1, 78) = 0.14	0.71	(1, 64) = 6.19	0.02
Exposure	(1, 78) = 0.0002	0.99	(1, 64) = 0.06	0.80
Dose by Age	(1, 78) = 0.62	0.43	(1, 64) = 0.89	0.35
Dose by Exposure	(1, 78) = 0.47	0.50	(1, 64) = 0.02	0.88
Age by Exposure	(1, 78) = 0.79	0.38	(1, 64) = 1.46	0.23
Dose by Age by Exposure	(1, 78) = 0.16	0.69	(1, 64) = 0.66	0.42

**Table 8:** Table for ANOVA results for blood ethanol concentrations at recovery of loss of righting reflex data.

BECs at Recovery of LORR	Female		Male	
	F Value	*p* Value	F Value	*p* Value
Dose	(1, 77) = 0.16	0.69	(1, 54) = 1.43	0.24
Age	(1, 77) = 3.81	0.055	(1, 54) = 3.99	0.05
Exposure	(1, 77) = 0.36	0.55	(1, 54) = 0.40	0.53
Dose by Age	(1, 77) = 0.0001	0.99	(1, 54) = 4.74	0.03
Dose by Exposure	(1, 77) = 0.61	0.44	(1, 54) = 2.91	0.09
Age by Exposure	(1, 77) = 0.012	0.91	(1, 54) = 0.17	0.68
Dose by Age by Exposure	(1, 77) = 1.76	0.69	(1, 54) = 1.04	0.42

**Table 9:** Table for ANOVA results for blood ethanol concentration time course analysis data.

BECs Time Course Analysis	Female		Male	
	F Value	*p* Value	F Value	*p* Value
Time	(1.6, 31.1) = 93.46	<0.0001	(2.2, 43) = 104.9	<0.0001
Age	(1, 21) = 9.51	0.006	(1, 21) = 5.65	0.03
Exposure	(1, 21) = 1.85	0.19	(1, 21) = 0.02	0.89
Time by Age	(3, 60) = 3.27	0.03	(3, 58) = 0.30	0.82
Time by Exposure	(3, 60) = 0.07	0.97	(3, 58) = 0.75	0.53
Age by Exposure	(1, 21) = 0.82	0.38	(1, 21) = 1.48	0.24
Time by Age by Exposure	(3, 60) = 0.90	0.45	(3, 58) = 0.12	0.95
